# Human GAPDH Is a Target of Aspirin’s Primary Metabolite Salicylic Acid and Its Derivatives

**DOI:** 10.1371/journal.pone.0143447

**Published:** 2015-11-25

**Authors:** Hyong Woo Choi, Miaoying Tian, Murli Manohar, Maged M. Harraz, Sang-Wook Park, Frank C. Schroeder, Solomon H. Snyder, Daniel F. Klessig

**Affiliations:** 1 Boyce Thompson Institute for Plant Research, Cornell University, 533 Tower Road, Ithaca, New York, 14853, United States of America; 2 The Solomon H. Snyder Department of Neuroscience, Johns Hopkins University School of Medicine, Baltimore, Maryland 21205, United States of America; 3 Department of Plant Pathology and Plant-Microbe Biology, Cornell University, Ithaca, New York, 14853, United States of America; UMBC, UNITED STATES

## Abstract

The plant hormone salicylic acid (SA) controls several physiological processes and is a key regulator of multiple levels of plant immunity. To decipher the mechanisms through which SA’s multiple physiological effects are mediated, particularly in immunity, two high-throughput screens were developed to identify SA-binding proteins (SABPs). Glyceraldehyde 3-Phosphate Dehydrogenase (GAPDH) from plants (*Arabidopsis thaliana*) was identified in these screens. Similar screens and subsequent analyses using SA analogs, in conjunction with either a photoaffinity labeling technique or surface plasmon resonance-based technology, established that human GAPDH (HsGAPDH) also binds SA. In addition to its central role in glycolysis, HsGAPDH participates in several pathological processes, including viral replication and neuronal cell death. The anti-Parkinson’s drug deprenyl has been shown to suppress nuclear translocation of HsGAPDH, an early step in cell death and the resulting cell death induced by the DNA alkylating agent N-methyl-N’-nitro-N-nitrosoguanidine. Here, we demonstrate that SA, which is the primary metabolite of aspirin (acetyl SA) and is likely responsible for many of its pharmacological effects, also suppresses nuclear translocation of HsGAPDH and cell death. Analysis of two synthetic SA derivatives and two classes of compounds from the Chinese medicinal herb *Glycyrrhiza foetida* (licorice), glycyrrhizin and the SA-derivatives amorfrutins, revealed that they not only appear to bind HsGAPDH more tightly than SA, but also exhibit a greater ability to suppress translocation of HsGAPDH to the nucleus and cell death.

## Introduction

Through most of human history, medicine has been based on plant remedies. Plant/herb-based medicine, sometimes referred to as traditional medicine (TM), still is the primary form of treatment for billions of people worldwide, particularly in developing countries, and is regaining favor in Western societies. Even in "modern" medicine, approximately half of the pharmaceuticals developed over the past 20 years and approved by the US FDA are natural products (mainly plant derived), or are synthetic derivatives of, or have at their core a prototype molecule derived from, natural products [1]. Salicylic acid (SA) and its derivatives, collectively termed salicylates, are a prime example. Acetyl SA, commonly called aspirin, has been the most widely used drug worldwide for the past two centuries—today Americans alone consume 80 million aspirin tablets daily. SA and its derivatives have been used for millennia to reduce pain, fever, and inflammation. Aspirin is also used to reduce the risk of heart attack, stroke, and certain cancers.

The primary action of aspirin in mammals has been attributed to the disruption of eicosanoid biosynthesis through the irreversible inhibition via acetylation of cyclooxygenases (COX) 1 and 2, thereby altering the levels of prostaglandins and leukotrienes [2]. However, since aspirin is rapidly de-acetylated by esterases in the bloodstream (t_1/2_ = 20 min), much of aspirin’s bioactivity can be attributed to its primary metabolite, SA, which reaches ~140 μM (~20 mg/L) within 1 h after intake of 500 mg of aspirin [3] and has a half-life of many hours in plasma [4]. Notably, although *in vitro* SA is a much weaker inhibitor of COX activity (IC_50_ >100 mg/L ~500 μM) than aspirin (IC_50_ = 6.3 mg/L ~35 μM), their anti-inflammatory effects are comparable *in vivo* [5,6]. Thus, there must be additional targets through which aspirin/SA brings about its many pharmacological effects.

In plants, SA is involved in many physiological processes including immunity, where it plays a central role [7,8]. Over the past quarter century, we have sought to elucidate SA’s mechanism(s) of action by identifying an array of plant proteins that bind SA, and, as a result, exhibit altered activity, which in turn modulates immune responses. Recently, we developed high-throughput screens to identify additional SA-binding proteins (SABPs) that may be targets of SA. This has led to the discovery of dozens of novel SA targets in plants [9–11].

To investigate whether aspirin has additional targets in humans, besides the cyclooxygenases, that may be responsible for some of aspirin’s multiple pharmacological activities, we employed similar screens of cultured human cells and identified many new potential targets of SA. In addition, several human proteins were identified as SA targets based on the SA-binding activity of their plant counterparts (orthologs). Among these is Glyceraldehyde 3-Phosphate Dehydrogenase (GAPDH).

GAPDH is a cytosolic homotetrameric enzyme that plays a central role in the production of energy, via a metabolic process called glycolysis. In addition to this housekeeping role, other functions of this protein have come to light in the past few decades, including its participation in DNA repair [12] and transcription [13]. It has RNA and DNA binding activities, which interestingly have been usurped by viruses for their replication, including hepatitis A, B, and C [14–17] and tomato bushy stunt virus [18]. In addition, numerous studies have linked GAPDH with cell death/apoptosis induced by a variety of methods such as treatment with N-methyl-(R)-salsolinol (a neurotoxin), staurosporine, MG132, reactive oxygen species (ROS) or N-methyl-N-nitroso-N^1^-nitroguanidine (MNNG, a DNA alkylating agent which mimics neuronal cell death induced by ROS; [19]) in multiple cell types including both neuronal and non-neuronal cells such as fibroblast and human embryonic kidney (HEK) 293 cells (reviewed in [20]). Thus, GAPDH is a major suspect in neurodegenerative diseases, including Alzheimer’s, Parkinson’s, and Huntington’s diseases [20]. The pioneering work of Ishitani and Chuang [21] and later Snyder and co-workers [19] revealed the central role GAPDH plays in neurodegeneration. Moreover, in 2006 Snyder and colleagues discovered that GAPDH, along with the ROS nitric oxide (NO) and the E3 ubiquitin ligase Siah, are involved in a novel cell death cascade. In brief, oxidative stress conditions lead to elevated levels of NO, which cause S-nitrosylation of GAPDH’s catalytic cysteine 152, which inactivates its glycolytic activity and induces its interaction with Siah. The monomeric GAPDH bound to Siah then is translocation to the nucleus, where it stabilizes this E3 ubiquitin ligase. This results in increased turnover of Siah’s nuclear target proteins leading to cell death. The anti-PD drug R-(-) deprenyl and related agent TCH346, which reduce neuronal cell death in both *in vitro* and *in vivo* models, prevent S-nitrosylation of GAPDH, block the GAPDH-Siah interaction, and inhibit GAPDH nuclear translocation [22].

Here we demonstrate that human GAPDH, like its plant counterpart, binds SA, as well as natural and synthetic derivatives of SA. Their binding suppresses both GAPDH nuclear translocation and cell death induced by MNNG.

## Materials and Methods

### Chemicals and anti-SA antibody

Salicylic acid (SA)(Cat#: 247588), Aspirin (Cat#: A5376) and Glycyrrhizin (Cat#: 50531) were purchased from Sigma-Aldrich. The synthesis of amorfrutin B1, FN1, and FN2 was described previously [23]. N-Methyl-N-nitroso-N'-nitroguanidine (MNNG) (Cat#: N-12560) was purchased from Chem Service Inc. Anti-SA antibodies were obtained from Acris Antibodies (Cat #: AP09810PU-L).

### Plasmid construction, expression and purification of His-tagged HsGAPDH

The plasmid pET28a-HsGAPDH used to express N-terminally His-tagged human (*Homo sapiens)* GAPDH (HsGAPDH) in *E*. *coli* was constructed by cloning the PCR-amplified protein-encoding sequence of human GAPDH (GenBank: M17851.1) into EcoRI and SalI sites of pET28a. The primers used are F: 5’- gcggaattcATGGGGAAGGTGAAGGTCGGAG -3’ and R: 5’- gcggtcgacTTACTCCTTGGAGGCCATGTGG -3’. Gene-specific sequences are in upper case and the restriction sites are underlined. cDNAs prepared from Hela cells were used for PCR amplification and cloning. The insert of the resultant plasmid was sequenced to confirm the accuracy. There was 1 nucleotide difference from the sequence in GenBank (M17851.1) without leading to any amino acid change. His-tagged recombinant HsGAPDH was expressed and purified in *E*. *coli* BL21(DE3) as described previously [24].

### SA-binding activity analysis of HsGAPDH

SA-binding activity analyses by photoaffinity labeling and surface plasmon resonance (SPR) were performed as described previously [9].

### Synthesis of N-acetyl 3-(2-aminoethyl) salicylic acid

Triethylamine (123 mg, 165 μL, 0.95 mmol) was added to a stirred solution of 3-(2-aminoethyl)salicylic acid (78 mg, 0.43 mmol) in a mixture of dimethylformamide (1.2 mL) and water (0.2 mL) at 0°C. After 5 min of stirring, a solution of acetic anhydride (46 mg, 42.5 μL, 0.45 mmol) in dimethylformamide (0.7 mL) was added dropwise with stirring over a time period of 5 min. After stirring at 20°C for 1 h, methanol (5 mL) was added and the mixture was evaporated to dryness in vacuo. The residue was chromatographed over silica using a Combiflash RF medium pressure automated chromatography system using a gradient of 5–40% methanol in dichloromethane as solvent. *N*-acetyl 3-(2-aminoethyl)salicylic acid was obtained as a colorless oil (purity >97% by ^1^H NMR spectroscopy) that solidified upon standing. Yield 45 mg (0.20 mmol).

^1^H NMR spectroscopy (methanol-d_4_, 600 MHz): δH 7.75 (dd, *J* = 8.0 Hz, 1.7 Hz, 1 H), 7.34 (dd, *J* = 7.4 Hz, 1.7 Hz, 1 H), 6.82 (dd, *J* = 8.0 Hz, 7.4 Hz, 1 H), 3.42 (t, *J* = 7.1 Hz, 2 H), 2.84 (dd, *J* = 7.1 Hz, 2 H), 1.99 (s, 3H). NMR spectroscopy was performed using a Varian INOVA 600 MHz NMR spectrometer (600 MHz ^1^H reference frequency) equipped with an HCN indirect-detection probe.

High-resolution negative-ion electrospray mass spectrometry: observed *m/*z 222.0781 [M + H]^-^, calculated for C_11_H_12_NO_4_^-^, 222.0772. High-resolution UHPLC- (ultra high pressure liquid chromatography-) mass spectrometry was performed on a Thermo Scientific-Dionex Ultimate3000 UHPLC system equipped with a diode array detector and connected to a Thermo Scientific Q Exactive Orbitrap mass spectrometer operated in electrospray negative (ESI-) ionization mode.

### Evaluation of the potential binding of SA derivatives by HsGAPDH

The potential binding between HsGAPDH and SA derivatives were evaluated by testing whether these derivatives were able to suppress the binding of HsGAPDH to 3AESA immobilized on CM5 sensor chip using SPR. The indicated concentrations of purified His-tagged HsGAPDH recombinant protein was mixed with 1 mM of various chemicals or the same amount of solvent, which were used to make chemical stock solution, in SPR running buffer [9], and then flown over 3AESA-immobilized CM5 sensor chip. The following data analysis was performed as determination of SA-binding activity using SPR [9].

### Nuclear fraction isolation

HEK 293 cells were pretreated with indicated concentrations of SA or other substances for 30 min then treated with 500 μM of N-Methyl-N-nitroso-N'-nitroguanidine (MNNG) for 30 min. Then the nuclear fraction was isolated as follows. The cells were scraped in ice cold hypotonic buffer containing 20 mM Tris-HCl, pH 7.4, 10 mM NaCl and 3 mM MgCl_2_. After a low speed (1,000 x g) spin for 5 min at 4°C, cells were resuspended in hypotonic buffer and incubated on ice for 15 min. NP-40 was then added to a final concentration of 0.5%. Nuclear fraction was pelleted by using a low speed (800 x g) spin for 10 min at 4°C. Nuclear fractionation using hypotonic buffer, NP-40 and centrifugation was repeated two more times as described above. Pellet was washed three times with ice cold phosphate buffer saline buffer and then resuspended in lysis buffer containing: 10 mM Tris, pH 7.4, 100 mM NaCl, 1 mM EDTA, 1 mM EGTA, 1 mM sodium fluoride (NaF), 20 mM Na_4_P_2_O_7_, 2 mM Na_3_VO_4_, 1% Triton X-100, 10% glycerol, 0.1% SDS, 0.5% deoxycholate, plus protease inhibitor cocktail containing; 0.5 μg/ml antipain, 1 μg/ml leupeptin, 1 μg/ml aprotinin, 1 μg/ml chymostatin and 1 μg/ml pepstatin A. The nuclear slate was cleared by centrifugation for 30 min at 10,000 x g at 4°C. Protein concentration was measured by the Bradford assay (Bio-Rad). Equal amounts of protein from nuclear extracts were resolved by 10% SDS-PAGE and then subjected to immunoblot analysis using anti-GAPDH antibody (GenScript USA Inc.; Cat #: A00192).

### Trypan blue exclusion test of cell viability

HEK 293 cells were pretreated with the indicated concentrations of salicylates or glycyrrhizin for 1 h before MNNG treatment. For MNNG treatment, cells were incubated in media containing 100 μM MNNG for 1 h and then the MNNG-containing medium was replaced with fresh medium containing the indicated concentrations of salicylates or glycyrrhizin without MNNG. Floating as well as adherent cells were harvested 6 h after MNNG treatment by 0.05% Trypsin-EDTA (Corning) and centrifugation for 5 min at 100 x g and then the resulting cell pellets were resuspended in serum-free DMEM medium. To determine the cell viability, the cells were stained with a trypan blue staining solution (GIBCO) according to manufacturer’s protocol, which stains dead cells. Briefly, diluted cell suspension was mixed with an equal volume of 0.4% trypan blue solution and then the number of unstained (viable) and stained (nonviable) cells were counted using hemocytometer.

## Results

### Human GAPDH binds SA and its derivatives

We previously developed a sensitive photoaffinity labeling approach using the photo-reactive SA analog 4-azido salicylic acid (4AzSA) as a probe to identify candidate SABPs (cSABPs) from Arabidopsis [9]. From a total of four pull-down assays, several dozen cSABPs were identified, including several members of the AtGAPDH family [10]. In independent experiments, HeLa cell extracts were subjected to affinity chromatography on a PharmaLink column to which SA had been linked as previously described [9–11]. Proteins that non-specifically bound the SA-linked matrix were removed using the biologically inactive SA analog, 4-hydroxybenzoic acid (4-HBA) in a stringent washing step. The affinity column was then competitively eluted with a high concentration of SA and the released proteins were identified by mass spectroscopy. HsGAPDH (GI:182861) was among the selected proteins.

**Fig 1:**
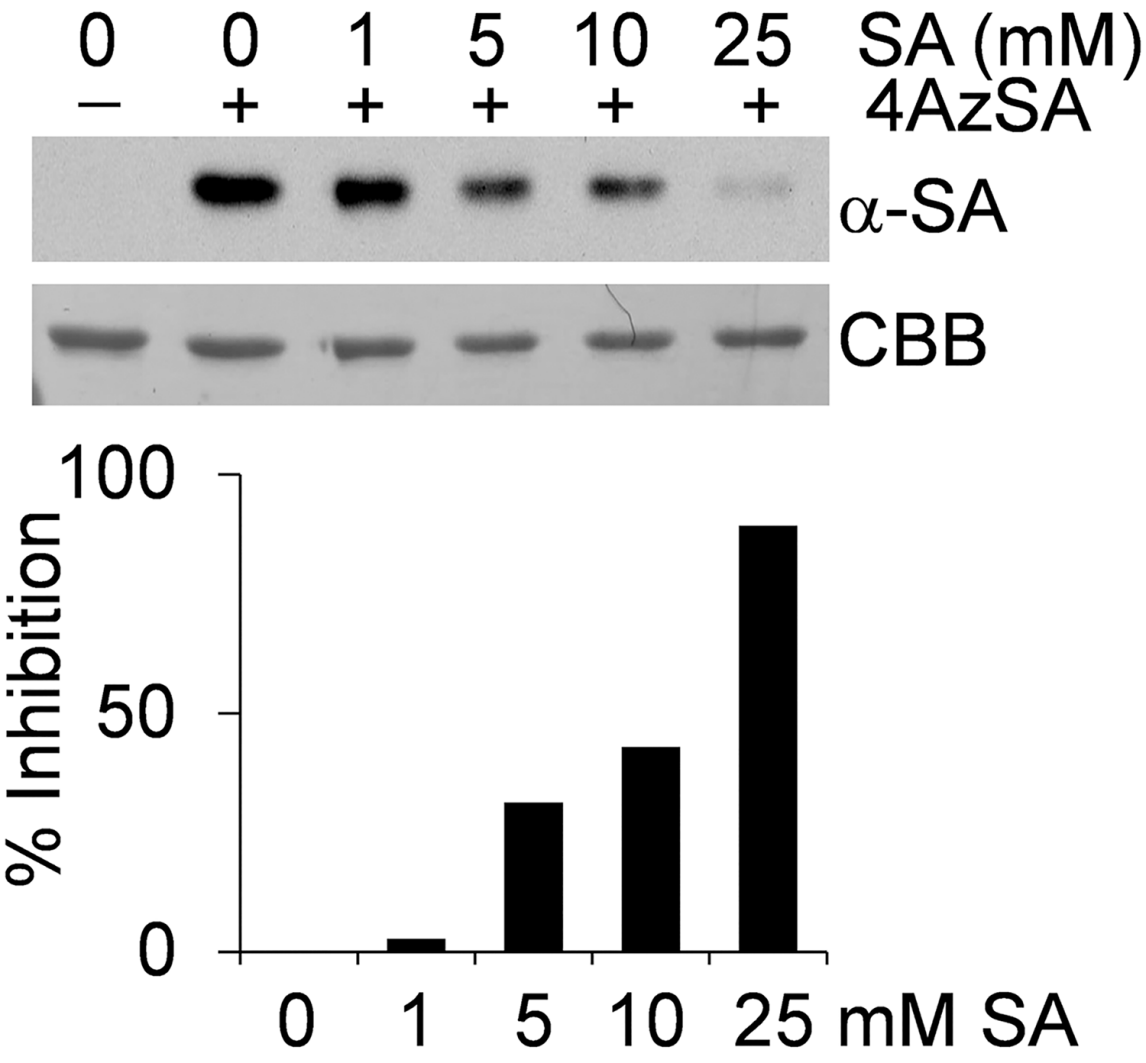
SA-binding activity of HsGAPDH monitored by photoaffinity labeling with 4AzSA. Crosslinking of 4AzSA to HsGAPDH is suppressed by SA. HsGAPDH was incubated without (-) or with (+) 4AzSA (0.05 mM) in the absence or presence of the indicated concentrations of SA for 1 h, and then exposed to UV light (50 mJ). Proteins crosslinked with 4AzSA were detected by immunoblot analysis using α-SA antibody. Proteins stained with Coomassie brilliant blue (CBB) served as a loading control. The experiment was repeated three times with similar results. Results are expressed as a percentage of inhibition (in lower panel) in the presence of the indicated concentrations of SA as compared to the signal observed in the absence of SA, which was assigned 0% inhibition.

To validate the SA-binding activity of HsGAPDH, its encoding gene was obtained; following expression as a His-tagged protein in *E*. *coli*, the recombinant protein was purified. Following our previous analyses of the AtGAPDHs [10], two sensitive approaches that utilize SA analogs in conjunction with either a photoaffinity labeling technique or a surface plasmon resonance (SPR)-based technology [9] were used to evaluate SA-binding activity. For the photoaffinity labeling approach, recombinant HsGAPDH was crosslinked with 4AzSA by UV irradiation in the absence or presence of SA, followed by immunoblot analysis with anti-SA antibody (Fig 1). Recombinant HsGAPDH crosslinked with 4AzSA, and this crosslinking was suppressed in the presence of increasing levels of SA. Prior analyses of the recombinant AtGAPDHs yielded similar results [10], arguing that the interaction between plant or human GAPDH and 4AzSA represents authentic SA-binding activity.

**Fig 2:**
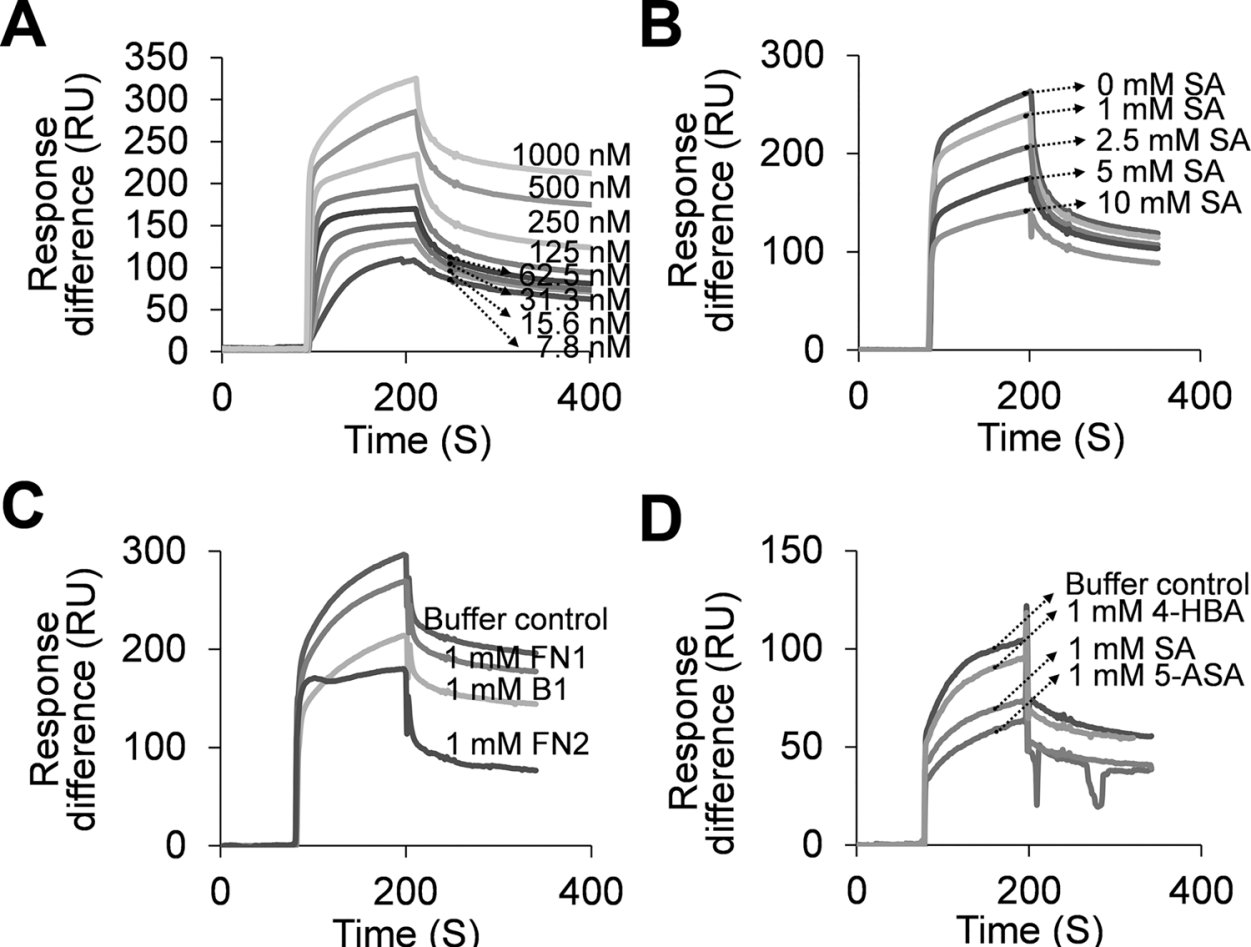
SA-binding activity of HsGAPDH monitored by SPR analyses. (A-D) Sensorgrams of HsGAPDH flowing over 3AESA-immobilized CM5 sensor chip surface. (A) Concentration-dependent response to increasing amounts of HsGAPDH. (B) Concentration-dependent inhibition by SA of HsGAPDH (0.5 μM) binding to a 3AESA-immobilized sensor chip. (C) Suppression by amorfrutin derivatives B1, FN1, and FN2 of HsGAPDH (0.5 μM) binding to a 3AESA-immobilized sensor chip. (D) Suppression by 4-HBA, SA and 5-ASA of HsGAPDH (0.27 μM) binding to a 3AESA-immobilized sensor chip. The signal from the mock-immobilized surface was subtracted.

To further confirm HsGAPDH’s SA-binding activity, we used SPR, which provides sensitive and quantitative measurements of bimolecular interactions in real time. An SA derivative, 3-aminoethyl SA (3AESA), was synthesized and affixed to the CM5 sensor chip via an amide bond formed between the amine group of 3AESA and the carboxyl groups on the chip. The ethylamine group was added at the 3 position of the phenyl ring because several SA derivatives with substitutions at this position retain the ability to bind plant SABPs and induce immune responses in plants [9]. When increasing concentrations of HsGAPDH were passed over the 3AESA-immobilized CM5 sensor chip surface, a corresponding increase in Response difference was observed (Fig 2A). HsGAPDH has high affinity for 3AESA, with an apparent K_d_ of 1.0 nmol/L (S1 Fig). Like the AtGAPDHs [10], binding to the sensor chip was suppressed by the presence of increasing levels of SA (Fig 2B). These findings further argue that binding of AtGAPDHs and HsGAPDH to the chip is a true reflection of their SA-binding activity.

**Fig 3:**
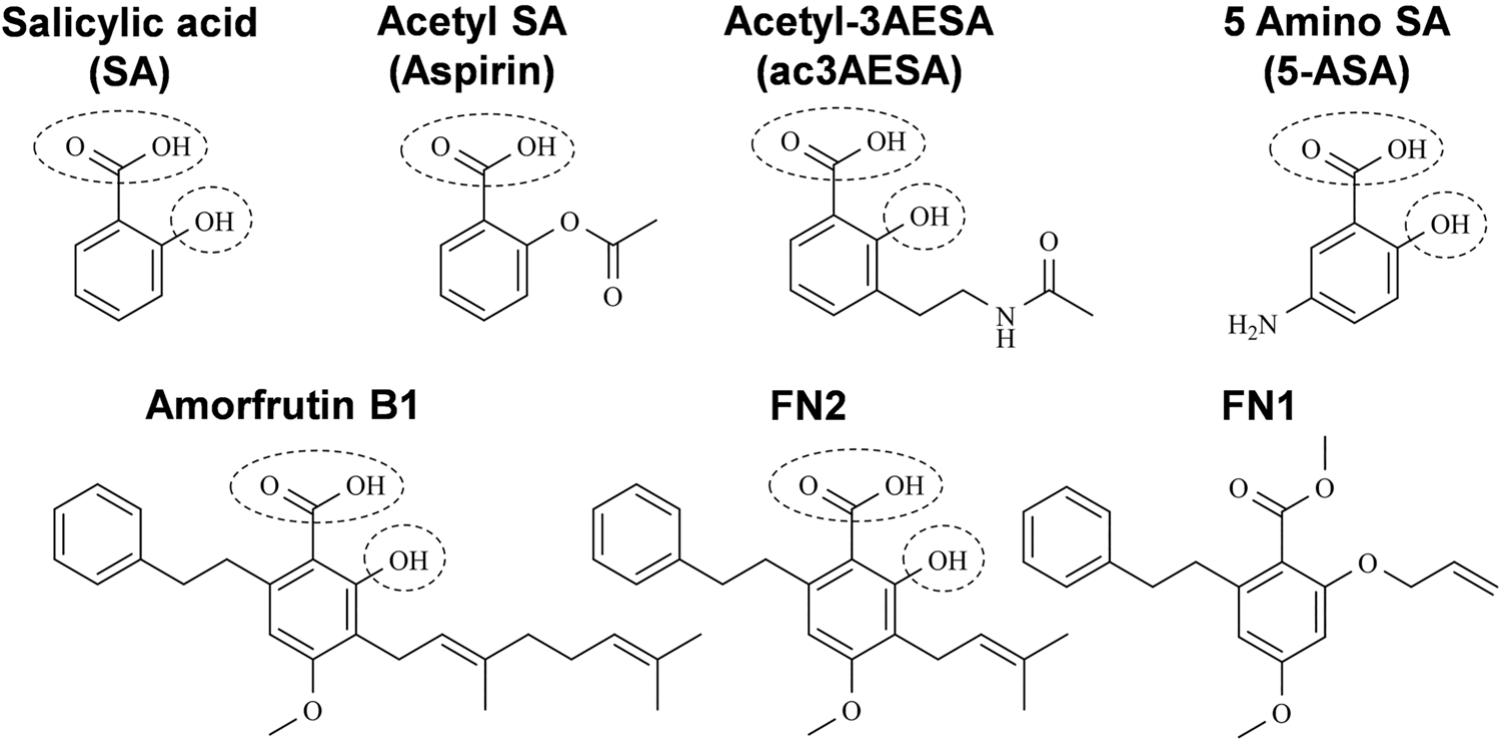
Chemical structures of salicylic acid and its synthetic and natural derivatives. Conserved hydroxyl (-OH) and a carboxyl (-COOH) groups of salicylates are denoted by dashed circles.

In a parallel study of High Mobility Group Box1 (HMGB1), which is another novel target of SA (aspirin) in humans [25], we identified the amorfrutins from the Chinese medicinal herb licorice (*Glycyrrhiza foetida*) as promising natural SA derivatives. Notably, amorfrutins were recently shown to have potent anti-diabetic activity, which was ascribed to their ability to bind to and activate the nuclear receptor peroxisome proliferator-activated receptor gamma [23]. Amorfrutins contain a phenyl ring with free carboxyl and hydroxyl groups at positions 1 and 2, respectively (Fig 3). This is the SA core structure, which we have found to be critical for the biological activity of SA derivatives in plants. The amorfrutins B1 and FN2 suppressed HsGAPDH binding to 3AESA immobilized on the sensor chip more effectively than SA [compare level of reduction of Response difference with 1 mM SA (Fig 2B) vs 1 mM B1 or FN2 (Fig 2C)]. In contrast, FN1, which is a synthetic derivative of B1 that contains neither the free carbonyl nor the hydroxyl group at positions 1 and 2, was a much weaker competitor than SA or the other two amorfrutins for binding to the immobilized 3AESA. Interestingly, both B1 and FN2, but not FN1, contain an alkyl group at position 3 (Fig 3). Given that 3AESA also contains an alkyl group at this site, these findings suggest that SA derivatives containing an alkyl group at position 3 may bind GAPDH more effectively than SA. To test this possibility, a new SA derivative, acetyl-3AESA (ac3AESA) was synthesized from 3AESA by converting the charged amino moiety to a neutral acetylated amide to resemble 3AESA linked to the SPR chip (Fig 3). Due to its limited solubility and stability in aqueous solution ac3AESA binding to HsGAPDH could not be assessed by SPR.

Interestingly, earlier studies have connected the SA derivative 5-amino SA (5-ASA) with HsGAPDH in Inflammatory Bowel Disease (IBD; [26–28]). In IBD, activated neutrophils infiltrating the inflammatory lesion produce potent ROS, including hydrogen peroxide, hypochlorite, and NO, which can inactivate HsGAPDH’s glycolytic activity. Hence, loss of HsGAPDH activity is used as a biomarker for IBD. 5-ASA, which is used to treat this disease, is an effective scavenger of ROS and its protective effect on HsGAPDH has been ascribed to this scavenging activity [28]. Since 5-ASA maintains the SA core, composed of the phenyl ring with free carboxyl and hydroxyl groups at positions 1 and 2, respectively (Fig 3), we tested whether it binds to HsGAPDH using SPR. 5-ASA suppressed the binding of HsGAPDH to 3AESA, arguing that it binds HsGAPDH (Fig 2D).

### SA and its derivatives suppress nuclear translocation of HsGAPDH

**Fig 4:**
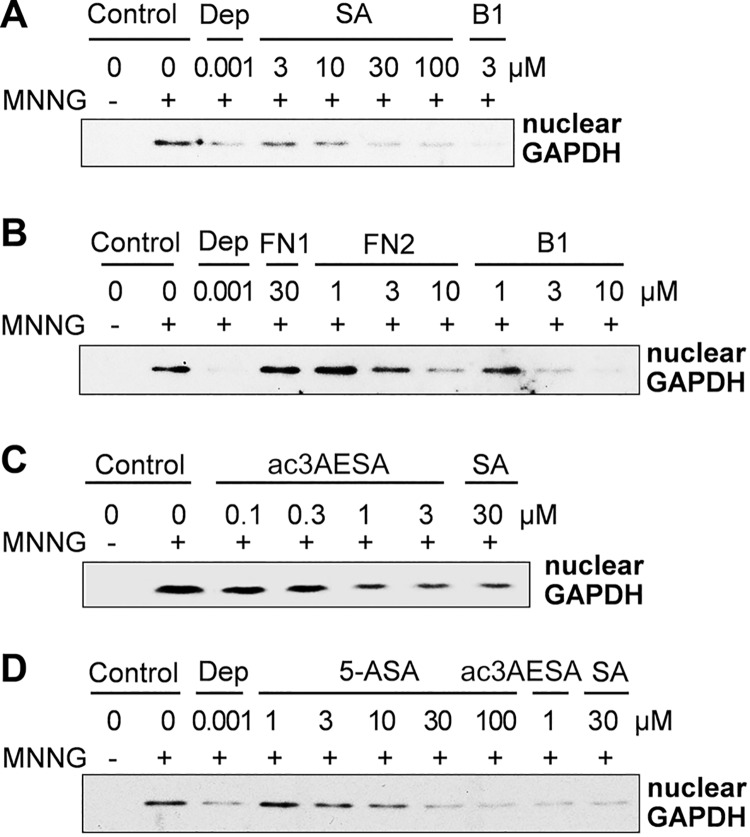
Effect of SA and its derivatives on MNNG-induced nuclear translocation of HsGAPDH in HEK 293 cells. (A-D) Suppression of MNNG-induced nuclear translocation of HsGAPDH by SA (A), by amorfrutin B1 and its derivatives FN1 and FN2 (B), by ac3AESA (C), and by 5-ASA (D). HEK-293 cells were treated with DNA alkylating agent MNNG and nuclear translocation of HsGAPDH in the absence and presence of SA and its derivatives was determined by immunoblotting the nuclear fraction with α-GAPDH antibody. The anti-Parkinson’s drug deprenyl (Dep) served as a positive control suppression of HsGAPDH nuclear translocation.

Given the involvement of HsGAPDH nuclear translocation in cell death and its association with neurodegeneration, we determined whether SA affects its nuclear translocation, the first step in cell death induced by a variety of agents including MNNG. As expected, MNNG treatment induced accumulation of HsGAPDH in the nucleus of HEK 293 cells (Fig 4A). Like the anti-Parkinson’s drug deprenyl, SA suppressed MNNG-induced nuclear translocation of HsGAPDH in a concentration-dependent manner. The ability of the natural and synthetic SA derivatives to suppress MNNG-induced HsGAPDH nuclear translocation was next assessed (Fig 4B–4D). Dose-response analyses indicated that B1 and FN2 were approximately 3–10 fold more efficacious than SA, while ac3AESA was approximately 30 fold more potent. Dose-dependent inhibition by 5-ASA was similar to that by SA (Fig 4D). By contrast, FN1 had no effect on MNNG-induced nuclear translocation of HsGAPDH (Fig 4B). Taken together, SA and its derivatives, which have free carboxyl and hydroxyl groups on the phenyl ring, effectively inhibited MNNG-induced nuclear accumulation of HsGAPDH. Moreover, the stronger inhibitory effects of these derivatives on nuclear translocation of HsGAPDH reflect the apparent higher affinity of these compounds for HsGAPDH.

### SA and it derivatives suppress cell death

**Fig 5:**
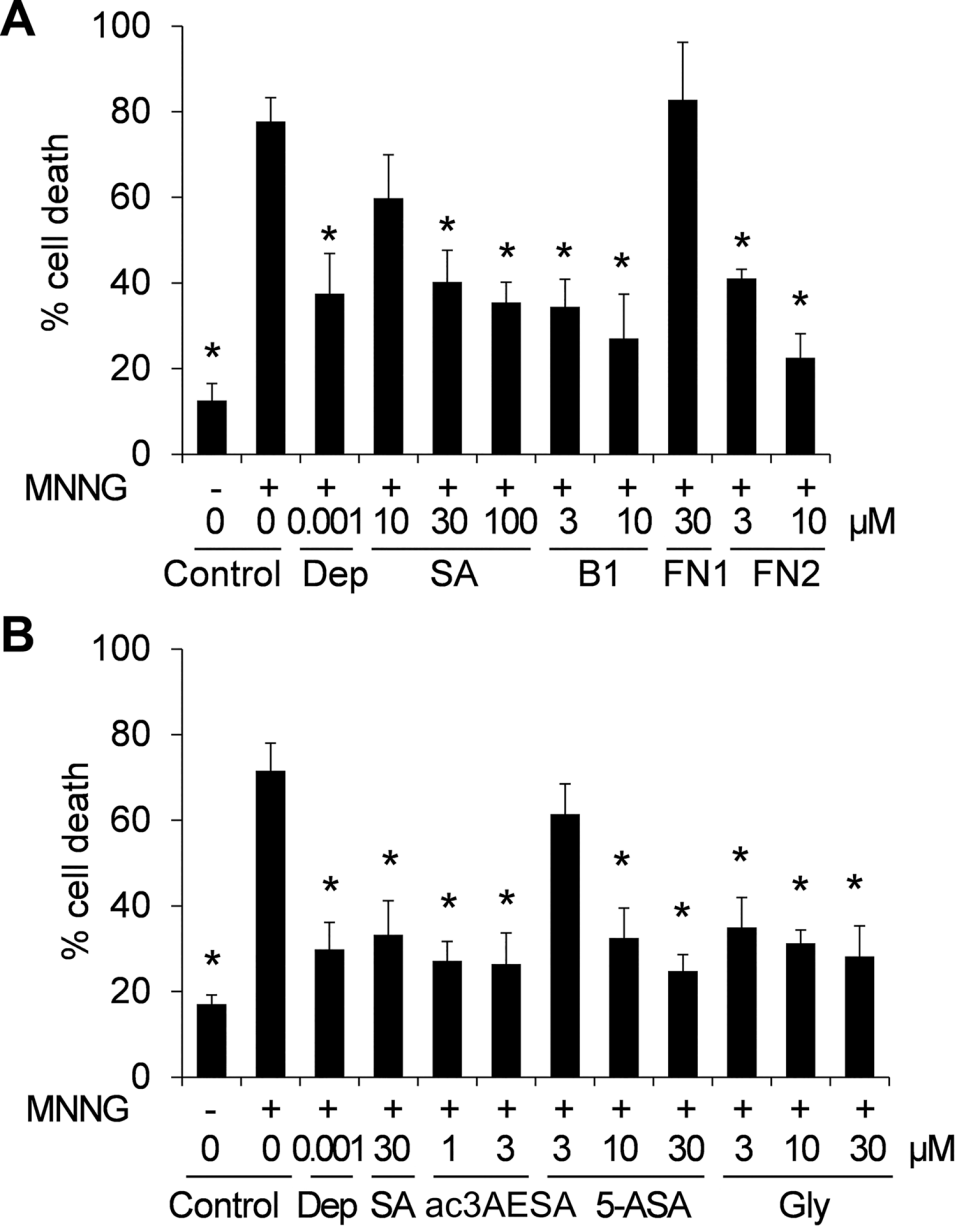
Effect of salicylates and glycyrrhizin on MNNG-induced cell death in HEK 293 cells. (A) Concentration-dependent inhibition of MNNG-induced cell death by SA, B1 and FN2. (B) Concentration-dependent inhibition of MNNG-induced cell death by ac3AESA, 5-ASA and Gly. The anti-Parkinson’s drug deprenyl (Dep) served as a positive control. Cell death was evaluated 6 h after MNNG treatment by trypan blue staining. Data are mean ± SD (n = 6). Asterisks indicate the statistical significance difference compared to the MNNG-treated control (one-way analyses of variance (ANOVA) with post-hoc t test; * P<0.05).

In addition to assessing whether SA and it derivatives suppress MNNG-induced nuclear translocation of HsGAPDH, their ability to suppress MNNG-induced cell death was determined. SA and the natural and synthetic derivatives that bound HsGAPDH and inhibited its nuclear translocation also suppressed cell death (Fig 5). Overall, the binding strength of these compounds exhibited for HsGAPDH correlated with their effectiveness at suppressing MNNG-induced HsGAPDH nuclear translocation and cell death. B1 and FN2 bound HsGAPDH more tightly than SA and reduced HsGAPDH nuclear translocation and cell death at lower concentrations than SA. 5-ASA, which bound HsGAPDH less tightly than B1 or FN2, inhibited these MNNG-induced responses to a lesser extent. Conversely, FN1, which bound HsGAPDH poorly, was ineffective at inhibiting HsGAPDH nuclear translocation or cell death. The most effective inhibitor of both HsGAPDH nuclear translocation and cell death was ac3AESA.

### A second natural product from *Glycyrrhiza foetida*, glycyrrhizin, binds HsGAPDH and suppresses MNNG-induced HsGAPDH nuclear translocation and cell death

**Fig 6:**
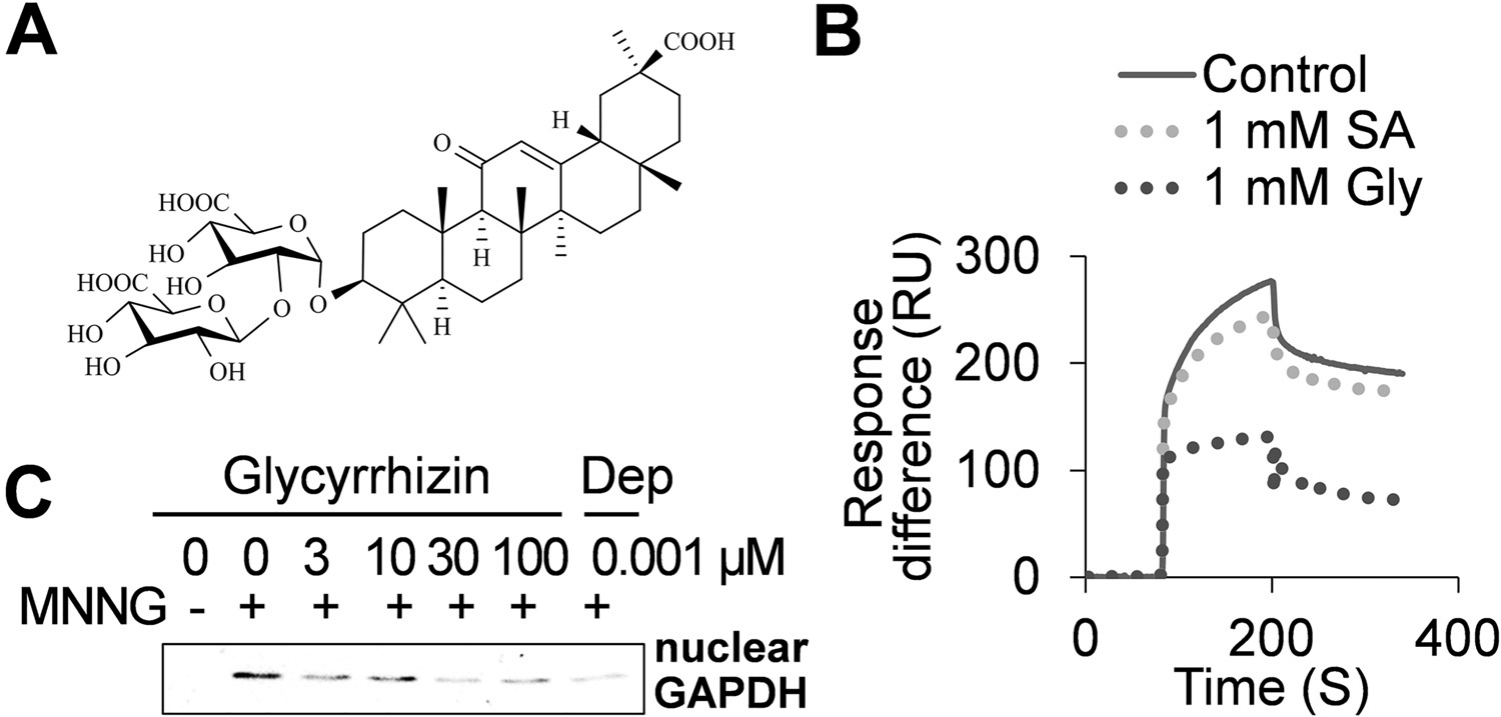
Effects of SA and glycyrrhizin on HsGAPDH binding to 3AESA and MNNG-induced nuclear translocation. (A) Chemical structure of glycyrrhizin. (B) SPR sensorgram of HsGAPDH (0.5 μM) in the absence (control) or presence of SA and glycyrrhizin flowing over 3AESA-immobilized CM5 sensor chip surface. The signal from the mock-immobilized surface was subtracted. (C) Suppression of nuclear translocation of HsGAPDH by glycyrrhizin. HEK-293 cells were treated with DNA alkylating agent MNNG and nuclear translocation of HsGAPDH in the absence and presence of glycyrrhizin was determined by immunoblotting the nuclear fraction with α-GAPDH antibody.

In addition to amorfrutins, *Glycyrrhiza foetida* produces a sweet-tasting compound called glycyrrhizin (Gly). Gly was previously shown to bind to HMGB1, thereby inhibiting its chemo-attractant activity [29], similar to SA [25]. Unexpectedly, the binding site in HMGB1 for Gly overlaps that of SA and its derivatives [25], despite their lack of structural similarity (Figs 3 and 6A). These results prompted us to test whether Gly also binds HsGAPDH. Gly was more effective than SA at competing with 3AESA for binding to HsGAPDH on the sensor chip (Fig 6B). Gly also suppressed MNNG-induced nuclear translocation of HsGAPDH (compare Fig 6C to Fig 4A) and cell death (Fig 5) more effectively than SA.

## Discussion

Several previous studies have demonstrated that high concentrations of salicylates (~10 mM) can induce apoptosis in a variety of cells [30–33]. For example, 10 mM sodium salicylate induced apoptosis in neutrophils by stimulating caspase-dependent turnover of Mcl-1, an anti-apoptotic member of the Bcl-2 family [33]. However, little was known about the effects of low concentrations of salicylate on apoptosis or cell death. Here we demonstrate that the low μM levels of SA and its natural and synthetic derivatives suppress cell death induced by MNNG, an agent which mimics neuronal cell death induced by ROS. This inhibition by these salicylates of cell death is likely due to their suppression of HsGAPDH nuclear translocation since it mirrors the effect of deprenyl. Deprenyl suppresses cell death by blocking HsGAPDH translocation to the nucleus [19]. Potentially therapeutic levels of SA for suppression of cell death are readily attainable since SA concentrations reaches ~140 μM (~20 mg/L) within 1 h following intake of 500 mg of aspirin [3]. Furthermore, the discovery of SA derivatives, which are 3–30 times more efficacious than SA, further supports the possibility that salicylates may be of therapeutic value in the treatment or even prevention of several widespread and devastating neurodegenerative diseases including Alzheimer’s and Parkinson’s diseases.

During the past half century, several targets of aspirin and its major metabolite SA have been identified. The most studied and celebrated are COX1 and COX2, whose enzymatic activities are irreversibly inhibited by aspirin’s acetylation of a serine in their catalytic site. This discovery by John Vane in the 1970’s [5] led to his selection for the Nobel Prize in Physiology or Medicine in 1982. More recently, additional potential targets have been uncovered, including Inhibitor of κB Kinase-β (IκK-β), whose kinase activity is inhibited by high concentrations of aspirin or SA [34]. IκK-β phosphorylates Inhibitor of κB, which leads to translocation of Nuclear Factor-κB (NF-κB) into the nucleus and the activation of NF-κB–regulated genes involved in inflammation, including cytokines. However, it is questionable whether IκK-β is a physiologically relevant target *in vivo* given that high concentrations of salicylates are needed to inhibit its kinase activity [35]. More recently, adenosine monophosphate-activated protein kinase (AMPK) was identified as an SA target [36]. High levels of salicylate (1 mM—30 mM) modestly increased the kinase activity of AMPK (1.5–2 fold) *in vitro* and *in vivo*. AMPK is a sensor of cellular energy levels, through which it regulates cell metabolism and growth; as such, it is involved in obesity and diabetes. In addition, supra-pharmacological concentrations SA (i.e., >5mM) have been shown to suppress phosphorylation, and hence inactivation, of retinoblastoma protein’s tumor suppression function, while inducing another tumor suppressor, p53 [35].

Using high-throughput screens that were developed to identify SABPs from plants [9–11], we have recently identified several novel SABPs from humans, including GAPDH and HMGB1, which was reported previously [25]. In contrast to all of the above human targets, SA suppresses the bioactivity of these two proteins in cells at low μM levels. Interestingly, these bioactivities are secondary, moonlighting functions of these two SABPs, some of which are shared by plant and animals. GAPDH in both plants and animals can be co-opted by some viruses to promote their replication, such as hepatitis A, B, C viruses in humans and tomato bushy stunt virus in plants [10,14–17]. Notably, SA interferes with the ability of plant GAPDH to bind the 3’ end of the minus strand of tomato bushy stunt viral RNA and thereby facilitate asymmetric replication [10]. Interestingly, Gly has anti-hepatitis virus activity and has been used in Japan for treatment of chronic hepatitis viral infection for decades [37–39]. Together, these observations suggest that SA or its more potent derivatives might also be useful for treatment of infection by this family of viruses.

The discovery that 5-ASA binds HsGAPDH and inhibits its translocation to the nucleus suggests that this compound may modulate IBD through more than one mechanism. In addition to 5-ASA’s purported role in scavenging ROS produced by infiltrating, activated neutrophils, its ability to bind and suppress HsGAPDH translocation to the nucleus may block induction of cell death. Since several natural and synthetic SA derivatives exhibit even stronger binding to HsGAPDH and/or greater efficacy in suppressing nuclear translocation of HsGAPDH and cell death than 5-ASA, it is possible that they will not only have therapeutic value in the treatment of IBD, but that they will be even more efficacious. It is worth noting that 5-ASA’s therapeutic effect on IBD may also involve another human target of SA/aspirin, HMGB1 [25]. Dying cell release HMGB1, which has potent pro-inflammatory activities that can be inhibited by SA, ac3AESA or amorfrutin B1 [25]. While we have not tested 5-ASA’s ability to bind to and thereby inhibit HMGB1’s pro-inflammatory activities, it seems highly likely given 5-ASA’s chemical structure and the results of our structure–activity analyses with HsGAPDH and HMGB1.

Although HsGAPDH binds 3AESA immobilized on a SPR sensor chip with high affinity (K_d_ = 1.0 nmol/L), suppression of this interaction required millimolar concentrations of SA (Fig 2B). Suppression of 4AzSA crosslinking to HsGAPDH also required high levels of SA (Fig 1). Similar results were obtained with HMGB1, SA’s other newly-identified target [25]. These results suggest that both proteins have relatively low affinity for SA *in vitro*, despite inhibition of their bioactivities *in vivo* at low μmol concentrations. While these results might suggest that SA is acting through another molecule(s), which modulates the bioactivities of these two proteins, rather than directly via SA binding to HsGAPDH or to HMGB1, we strongly suspect that SA’s effect on HsGAPDH bioactivities is direct for the following reasons. First, we have rigorously established that HMGB1 is the direct target of SA using a mutant of HMGB1 (R24A/K28) whose SA-binding site was altered. This mutant protein retains its chemo-attractant activity, but this activity is no longer inhibitable by SA, because the mutant can no longer bind SA. Second, as with HMGB1, SA derivatives that bind to HsGAPDH more tightly than SA, also are stronger inhibitors of HsGAPDH nuclear translocation and cell death. Third, Gly, which appears to act as a mimic of SA both with HsGAPDH and with HMGB1, in which its binding site and that of SA overlap, has higher affinity for HsGAPDH than SA and is a stronger inhibitor of HsGAPDH nuclear translocation and cell death. Together the results with HsGAPDH (and with HMGB1) argue that SA’s effect on HsGAPDH nuclear translocation and cell death is due to its direct interaction with HsGAPDH. However, this has not been rigorously established using genetics. We suspect that *in vivo* the conformations of HsGAPDH (and of HMGB1) are modified by the interaction with other molecules, so that the binding of salicylates is facilitated. These “missing molecules” have yet to be identified.

## Conclusion

Taken together, the identification of HsGAPDH, as well as HMGB1, as targets for SA/aspirin in humans, coupled with a greater understanding of how salicylates regulate the activities of these proteins, has the potential to open broad new avenues in the treatment of a wide variety of diseases. Moreover, the identification of synthetic and natural derivatives of SA which are more potent inhibitors than SA of both HsGAPDH’s and HMGB1’s disease-associated bioactivities provides proof-of-concept that new SA-based molecules with high efficacy are attainable.

## Supporting Information

S1 Fig Analysis of salicylic acid (SA)-binding activity of HsGAPDH by surface plasmon resonance. Sensorgrams of concentration-dependent HsGAPDH interacting with 3AESA immobilized on the SPR sensor chip. HsGAPDH has strong affinity for 3AESA with an apparent Kd of 1.01 nmol/L.
(TIF)
