# Supplementary figures and images for: Human GAPDH Is a Target of Aspirin’s Primary Metabolite Salicylic Acid and Its Derivatives
Source: PLoS One. 2015 Nov 25;10(11):e0143447. doi: 10.1371/journal.pone.0143447 (PMC4659538; doi:10.1371/journal.pone.0143447)

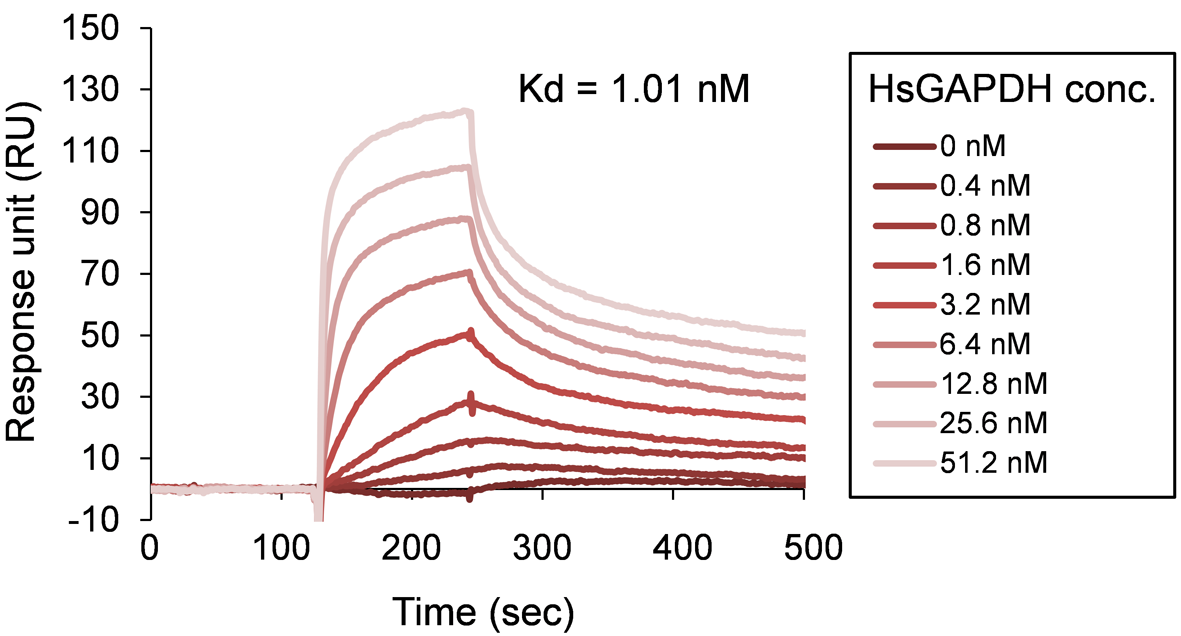

Supplement: S1 Fig — Sensorgrams of concentration-dependent HsGAPDH interacting with 3AESA immobilized on the SPR sensor chip. HsGAPDH has strong affinity for 3AESA with an apparent Kd of 1.01 nmol/L. (TIF) [file pone.0143447.s001.tif]
